# Elicited ROS Scavenging Activity, Photoprotective, and Wound-Healing Properties of Collagen-Derived Peptides from the Marine Sponge *Chondrosia reniformis*

**DOI:** 10.3390/md16120465

**Published:** 2018-11-23

**Authors:** Marina Pozzolini, Enrico Millo, Caterina Oliveri, Serena Mirata, Annalisa Salis, Gianluca Damonte, Maria Arkel, Sonia Scarfì

**Affiliations:** 1 Department of Earth, Environment and Life Sciences (DISTAV), University of Genova, Via Pastore 3, 16132 Genova, Italy; caterina.oliveri@unige.it (C.O.); serenamira94@gmail.com (S.M.); soniascarfi@unige.it (S.S.); 2 Department of Experimental Medicine (DIMES), Biochemistry Section, University of Genova, Viale Benedetto XV 1, 16132 Genova, Italy; enrico.millo@unige.it (E.M.); annalisa.salis@unige.it (A.S.); gianluca.damonte@unige.it (G.D.); mariaarkel27@gmail.com (M.A.); 3 Centre of Excellence for Biomedical Research (CEBR), University of Genova, Viale Benedetto XV 9, 16132 Genova, Italy; 4 Inter-University Center for the Promotion of the 3Rs Principles in Teaching & Research (Centro 3R), 56122 Pisa, Italy

**Keywords:** marine collagen peptide, collagen hydrolysates, antioxidant, cosmetics, inflammation

## Abstract

Recently, the bioactive properties of marine collagen and marine collagen hydrolysates have been demonstrated. Although there is some literature assessing the general chemical features and biocompatibility of collagen extracts from marine sponges, no data are available on the biological effects of sponge collagen hydrolysates for biomedical and/or cosmetic purposes. Here, we studied the in vitro toxicity, antioxidant, wound-healing, and photoprotective properties of four HPLC-purified fractions of trypsin-digested collagen extracts—marine collagen hydrolysates (MCHs)—from the marine sponge *C. reniformis*. The results showed that the four MCHs have no degree of toxicity on the cell lines analyzed; conversely, they were able to stimulate cell growth. They showed a significant antioxidant activity both in cell-free assays as well as in H_2_O_2_ or quartz-stimulated macrophages, going from 23% to 60% of reactive oxygen species (ROS) scavenging activity for the four MCHs. Finally, an in vitro wound-healing test was performed with fibroblasts and keratinocytes, and the survival of both cells was evaluated after UV radiation. In both experiments, MCHs showed significant results, increasing the proliferation speed and protecting from UV-induced cell death. Overall, these data open the way to the use of *C. reniformis* MCHs in drug and cosmetic formulations for damaged or photoaged skin repair.

## 1. Introduction

Collagens constitute a variegated family of structural proteins that are usually found in the extracellular matrix (ECM) of many tissues in multicellular organisms. Here, they participate in the formation of a complex glycosaminoglycan/protein network ensuing the structural support and physiological integrity of the tissues, mainly, but not exclusively, of mesodermal origin. Thanks to their low immunogenicity across the species and the remarkable mechanical and/or bioactive properties, their collagen extracts, derived gelatins, and peptide hydrolysates are frequently used in health-related sectors [1], cosmetics, and the food industry [2].

Collagen and collagen derivatives are mainly obtained from porcine and bovine skin and bones. Unfortunately, in the last years, the increased risk of BSE and TSE (bovine spongiform encephalopathy and transmissible spongiform encephalopathy, respectively) human infections from cows and pigs, as well as religious constraints on use of porcine derivatives, has led to the investigation of new possible animal sources of collagen. The most intriguing and promising ones come from the marine environment [3]. These collagens are mainly extracted and processed from the waste of fish and molluscs in the fishing industry [4,5], as well as from other invertebrates that are particularly abundant in the marine environment and rich in collagen, such as jellyfishes or sponges [6,7,8]. Indeed, the successful use of marine collagens from fish, echinoderms, molluscs, and jellyfish populations in human health-related applications such as the evaluation of biological compatibility and use in regenerative medicine, wound-healing, and cosmetics has been increasingly reported [9,10,11,12,13].

Over the years, several studies have also been performed to evaluate the biocompatibility and regenerative medicine potential of marine sponge-derived collagen extracts [8,14,15]. The phylum Porifera is the most ancient metazoan group still thriving on our planet. These are very simple, sessile animals, lacking any real tissue or organ, and formed only by few specialized cell types embedded in a complex ECM network that is very rich in collagen [16,17,18]. In particular, one of the most described collagens in this phylum derives from the demosponge *Chondrosia reniformis*; indeed, in this animal, it displays peculiar physicochemical characteristics and dynamic plasticity [19,20]. Quite recently, some collagen gene sequences, as well as that of a collagen maturation enzyme, have been uncovered in this animal [21,22,23]. Furthermore, *C. reniformis* collagen has also demonstrated its utility as a carrier in form of nanoparticles and as a coating for drug preparations, and its lack of toxicity on human skin has been assessed [24,25,26]. Recently, also, the possibility of using it in the form of thin biocompatible membranes for tissue engineering and regenerative medicine purposes has been evaluated [7].

Overall, the high biotechnological potential of sponges has been clearly recognized in the last years due also to the noteworthy production of bioactive secondary metabolites [27]. Indeed, these molecules have arisen the interest of the pharmacological industry. Thus, in order to obtain commercial quantities of the compounds of interest, various mariculture systems [28,29] have been developed for the full exploitation of the pharmacological potential of these organisms. In this view, bioactive compound extraction from sponge aquacultures would lead to the waste of sponge biomass by-products that could be further employed for the extraction of marine collagen. Furthermore, the great biodiversity in the phylum Porifera may give rise, depending on the species exploited, to a wide variety of different sponge biomasses suitable for different applications, alternatively privileging collagen and/or silica-producing sponges [30]

In addition to the pharmacological and cosmetic use of marine collagens and gelatins from various animal sources per se, also, the employ of bioactive peptides derived from controlled collagen enzymatic hydrolysis has been increasingly reported. Indeed, the potentialities both in the nutraceutical field as well as in vitro, in vivo, and eventually in clinical studies have been evaluated, demonstrating the significant positive effects of collagen hydrolysates both in physiological conditions as well as in ill health [31].

Indeed, a plethora of biological activities have been ascertained by the use of marine collagen-derived peptides such as antimicrobial, antihypertensive, antidiabetic, opioid, calciotropic, secretagogue, joint and bone-regenerative, antioxidant, wound-healing, UV-protective, and antityrosinase activities, both in vitro and/or in vivo, alternatively administered orally or systemically or even in topical concoctions [11,31]. In the years, particular attention has been given to the antioxidant properties of these peptides, since excess of intracellular reactive oxygen species (ROS) has been linked to the development and chronicization of many pathological conditions such as cardiovascular, neurodegenerative, inflammatory, cancer, and age-related illnesses [32,33,34]. Thus, the search for new molecules with antioxidant activity, especially from natural sources, is continuously pursued as potential drugs for many pathological conditions. In this regard, a significant antioxidant activity has been demonstrated in collagen hydrolysates from different invertebrates such squid and jellyfishes [35,36], as well as from many fishes, such as cod, cobia, Nile tilapia, tuna, and sole skin [35,37,38,39,40] to cite a few. Another important field of studies where marine collagen hydrolysates have proven valuable results is related to skin repair, regeneration, and aging [11]. Skin is a physical and chemical barrier of the body against harmful foreign events and pollutants. Indeed, environmental attacks under the form of chemicals, ultraviolet (UV) light, mechanical injuries, and temperature changes may cause serious damage to this important natural barrier [41]. In recent years, a considerable amount of attention has been given to the use of marine collagen hydrolysates as skin repairing/regenerating agents in nutraceutical, pharmacological, and cosmetic formulations in different pre-clinical and clinical studies. Collagen hydrolysates from various sources have demonstrated good biocompatibility, penetration ability, and skin-protective properties in different experimental contexts. As a few examples, in pre-clinical studies, collagen hydrolysates from jellyfish, salmon, and Pacific cod skin have proven significant protective effects on photoaging in vivo [42,43,44], while collagen peptides from Nile tilapia and Chum salmon skin have demonstrated wound-healing properties [40,45]. Last but not least, clinical studies have also shown improved skin aging parameters after the oral administration of fish collagen peptides [46], definitely indicating the plausibility of exploiting the bioactive properties of marine collagen hydrolysates in many physiopathological conditions of the skin.

Although the bioactive properties of several natural peptides extracted from various sponges have already been reported [47], to date, no information is available on the antioxidant and skin-healing properties of collagen hydrolysates derived from sponges. Indeed, the only available data are on the cosmetic use of *C. reniformis* (non-digested) collagen extracts in cosmetic formulations to assess their biocompatibility and use in substitution to conventional collagens from mammals, which proved successful [24]. Thus, starting from the above-mentioned observations of *C. reniformis* collagen biocompatibility in vivo, the aim of the present work was to produce trypsin-derived *C. reniformis* collagen hydrolysates and explore their performance as antioxidant, UV-protecting, and wound-healing molecules. To this purpose, four fractions of marine collagen hydrolysates (MCHs) from *C. reniformis* were obtained by the enzymatic digestion of sponge collagen extracts followed by reverse-phase HPLC purification, and subsequently their ROS scavenging activity in cell-free tests as well as in an in vitro macrophage model of cell inflammation was investigated. Conversely, the ability to stimulate collagen production in a fibroblast cell line and protect from UV-induced cell damage both in a fibroblast and in a keratinocyte cell line was assessed; the wound-healing properties were also demonstrated by the well-known in vitro “scratch test”.

## 2. Results and Discussion

### 2.1. Marine Collagen Hydrolysate (MCHs) Purification Yield and Chemical Features

**Figure 1:**
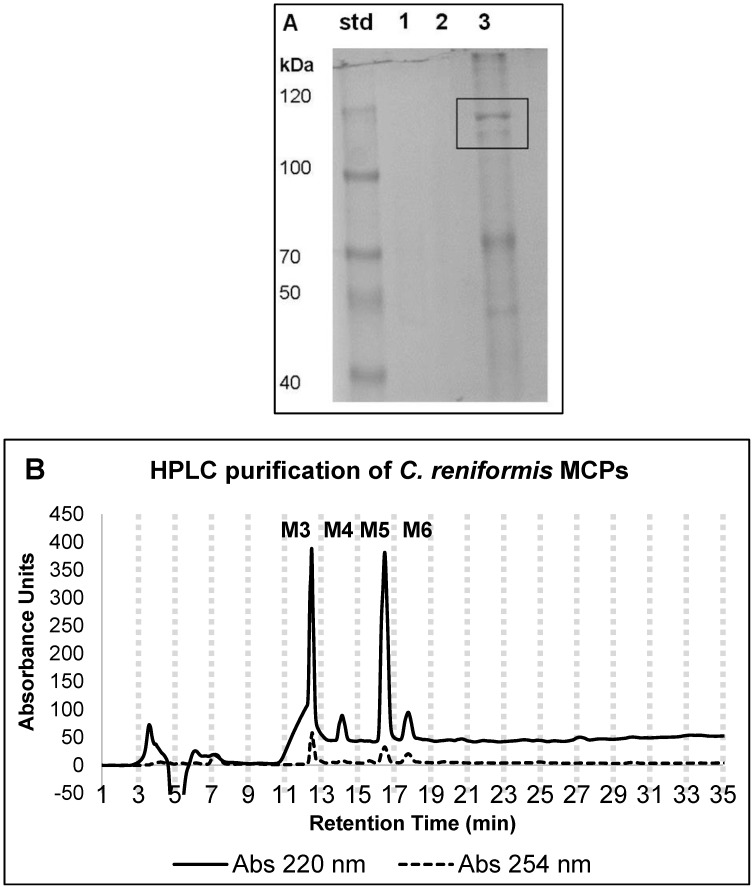
Characterization and purification of *C. reniformis* marine collagen hydrolysates (MCHs). (**A**) SDS-PAGE analysis of undigested and digested sponge collagen. *C. reniformis* trypsin-digested collagen solutions, two different preparations (lane 1 and 2), and undigested collagen suspension (lane 3) were analyzed on 7.5% SDS polyacrylamide gel and Coomassie blue stained. std = standard molecular weight markers. In lane 3, highlighted in the box, α1-chain and α2-chain of fibrillar collagen. (**B**) RP-HPLC (reversed phase high-performance liquid chromatography) profile of the *C. reniformis* trypsin-digested collagen solution in the chromatography used to obtain the MCH fractions. During the purification, fractions were collected every two minutes, as indicated by the vertical dotted grey lines on the chromatogram. The fractions of interest are indicated by the abbreviations M3, M4, M5, and M6, respectively. The analytical conditions are reported in the Materials and Methods (Section 4.4). The continuous line indicates the chromatogram registered at 220 nm, while the dotted line indicates the chromatogram of the same run at 254 nm.

**Figure 2:**
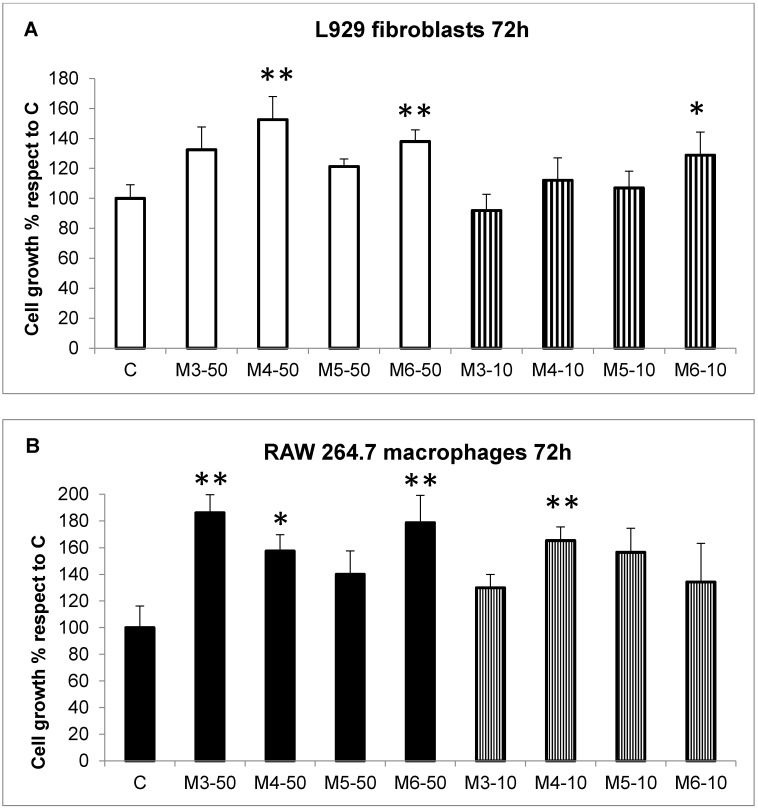

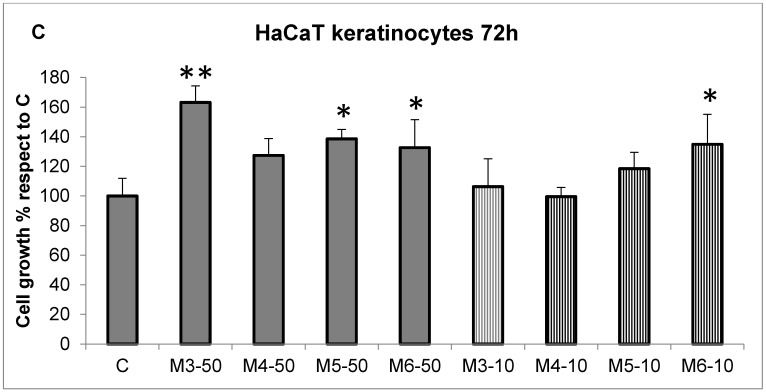
Cell toxicity evaluation. (**A**) L929 fibroblast cell growth quantitative evaluation, by the cell viability MTT test at 72 h, in the presence or absence of the four different MCHs (M3–M6) at the concentration of 50 g/mL (white bars) and 10 g/mL (striped bars). Results are expressed as cell percentages with respect to controls, and are the mean ± S.D. of three experiments performed in quadruplicate. Asterisks indicate the significance in a paired Tukey test (ANOVA, *p* < 0.0005; Tukey vs. C: * *p* < 0.05, ** *p* < 0.005, respectively). (**B**) RAW 264.7 macrophages cell growth quantitative evaluation, in the same conditions as (A). Black bars: MCH fractions 50 g/mL, striped bars: MCH fractions 10 g/mL. Results are expressed as cell percentages with respect to controls, and are the mean ± S.D. of three experiments performed in quadruplicate. Asterisks indicate significance in a paired Tukey test (ANOVA, *p* < 0.005; Tukey vs. C: * *p* < 0.05, ** *p* < 0.01, respectively). (**C**) HaCaT keratinocytes cell growth quantitative evaluation, in the same conditions as (A). Grey bars: MCH fractions 50 g/mL, striped bars: MCH fractions 10 g/mL. Results are expressed as cell percentages with respect to controls, and are the mean ± S.D. of three experiments performed in quadruplicate. Asterisks indicate significance in paired Tukey test (ANOVA, *p* < 0.00005; Tukey vs. C: * *p* < 0.05, ** *p* < 0.0005, respectively).

Initially, the yield of total protein content in the *C. reniformis* sponge collagen suspension (before the enzymatic digestion to obtain MCHs) was evaluated, giving a value of 1.2 g ± 0.17 of proteins from 25 g of wet sponge tissue (starting material). Following 18 h of trypsin digestion at 37 °C, the viscous collagen suspension that resulted was only partially solubilized. After enzyme inactivation and sample centrifugation, the supernatant containing the MCH solution mixture had the aspect of a clear, dark-colored solution. An initial characterization of the MCH clear solution mixture was obtained by evaluating the hydrolysis degree (HD) after the trypsin digestion procedure. The value was calculated as the percentage of the amino acid content of the MCH clear solution mixture with respect to the amino acid content of the undigested suspension, and resulted in 53.5 ± 7.1% protein total digestion. Then, to obtain a raw evaluation of the peptide sizes in the MCH solution mixture, an SDS gel electrophoresis was performed on the undigested, and trypsin-digested, collagen suspensions (Figure 1A). In particular, the electrophoretic analysis of a 7.5% polyacrylamide gel of the undigested collagen suspension (lane 3) revealed the presence of two bands of about 100 kDa corresponding to α1-fibrillar and α2-fibrillar collagen chains, and one band at 70 kDa that likely could correspond to the non-fibrillar collagen, which was identified, characterized, and obtained in recombinant form by some of us [21,22,23,48]. Conversely, in the trypsin-digested MCH solution mixture (lane 1 and lane 2, two different digestions), no protein bands were observable, indicating that in the established enzymatic conditions, a good degree of collagen digestion, and release of peptides with a molecular average size lower than 40 kDa, was obtained. The trypsin-digested collagen solution was then purified by preparative reversed phase high-performance liquid chromatography to obtain different fractions of MCHs. The Figure 2B shows a comparative analysis of the RP-HPLC profile at λ = 220 and 254 nm of the MCH mixture. The fractions were collected every two minutes starting from minute seven until minute 21; then, the fractions deriving from three different HPLC runs were concentrated under vacuum and repeatedly lyophilized to remove formic acid before suspension in water. In preliminary experiments (not shown), all of the fractions were analyzed for their cytotoxicity and their antioxidant activity by the DPPH assay, but only four fractions, which were indicated as M3, M4, M5, and M6 in Figure 1B, showed activity. As such, only these four fractions were used to perform all of the following experiments to evaluate the biological activity of the *C. reniformis* MCHs. The characterization experiments throughout the paper are the mean of two different extractions from different sponges. HPLC spectra were very similar from one preparation to the other (not shown); thus, we can say that the extraction procedure and the enzymatic digestion giving the MCH mixture were quite reproducible.

A first attempt of the chemical characterization of the four MCH fractions by HPLC coupled to electrospray mass spectrometry has been made, giving us indications that the peptides obtained are not attributable to simple amino acid chains from which, by MS/MS (tandem Mass Spectrometry) analysis, it would be possible to reveal the sequences (data not shown). Preliminary MS/MS experiments did not obtain any conclusive data. This could be due to the presence of PTMs (post-translational modifications). It is well-known that collagen from sponges, and from *C. reniformis* in particular, undergoes significant glycosylation [49], which could impair a direct MS identification. In addition, the widespread presence in collagen of proline and lysine and their hydroxylated forms further complicates the enzymatic cleavage and subsequent characterization. Supplementary and more specific analyses will help us in the future shed light on the peptide composition of the most interesting MCH fractions.

Finally, to obtain a measure of the fractions of peptides solely deriving from the sponge collagen digestion and not from other extracellular matrix sponge proteins co-purified with collagen, the hydroxyproline content was evaluated by the chloramine-T method. Starting from one mg/mL of undigested collagen suspension, the amount of the cyclic amino acid was of 83.21 ± 12.3 µg/mL, while in the same amount of MCH clear solution mixture, it was of 109.99 ± 4.78 µg/mL. Thus, the percentage of this amino acid with respect to the total protein content is 8.3% and 10.9% for the two solutions respectively, and since its presence in marine collagen proteins is usually in the range of 10%, we can conclude that both the collagen suspension as well as the digested MCH solution are mostly composed of collagen or collagen derivatives. Finally, in the HPLC-purified MCH fractions, the concentration of hydroxyproline content ranged from a minimum of ≈7 µg/mL in an M3 fraction to a maximum of ≈37 µg/mL in an M5 fraction (Table 1). These data confirm the presence of collagen-derived peptides in all four MCH fractions, although they also indicate the presence of variable moieties of peptides directly deriving from the digestion of collagen domains containing the cyclic amino acid in the various fractions. In particular, the M4 and M5 fractions that resulted were particularly enriched in collagen-derived peptides containing hydroxylproline (≈ four and five times higher than the less performant M3 fraction, respectively), and were probably the most promising for the biological activities that were subsequently tested.

### 2.2. Effect of the MCP Fractions on Cell Growth of Specific Cell Lines

The biocompatibility and lack of toxicity of collagen extracts from *C. reniformis* on human skin have already been demonstrated [24], as well as their use as biocompatible material/scaffold for cell adhesion/proliferation and regenerative medicine purposes [7]. This opens the door also to the possible use of *C. reniformis* collagen derivatives for human health purposes, although the utility and feasibility of the latter has to be demonstrated. In fact, to our knowledge, no studies on the biocompatibility and biological effects of collagen hydrolysates obtained from marine sponges have ever been performed. Thus, the four MCH fractions obtained by the HPLC separation of *C. reniformis* collagen suspensions were initially analyzed in vitro on different cell types for their effects on cell growth and toxicity. The immortalized cell lines were chosen for the further evaluation of the specific biological activities of the MCH fractions on each cell line, namely the stimulation of collagen production and release in fibroblasts, ROS scavenging activity in activated macrophages, and photoprotective and wound-healing properties in keratinocytes and fibroblasts. Thus, the cell lines that were utilized in our experiments were the collagen-producing L929 murine fibroblasts, the activated-ROS producing RAW 264.7 murine macrophages, and the photosensitive HaCaT human keratinocytes. Cells were treated for 72 h with the four MCH fractions (M3 to M6) at the final concentrations of 50 µg/mL and 10 µg/mL (Figure 2, panels A–C, full colored bars and striped bars, respectively), and then analyzed by the MTT (3-(4,5-dimethylthiazol-2-yl)-2,5-diphenyltetrazolium bromide) cytotoxicity test. At both concentrations, no cytotoxicity was ever observable for all of the MCH fractions that were used, nor in the three cell lines tested, with respect to untreated cells (C). Surprisingly, a slight cell growth stimulation was measured in all of the cell lines, even if with different MCH fractions. In particular, in L929 fibroblasts (panel A), a slight but significant increase of cell number after 72 h was observed with M4 and M6 MCH fractions (1.52 ± 0.155 fold increase for M4-50; 1.38 ± 0.077 and 1.29 ± 0.015 fold increase for M6-50 and M6-10, respectively, compared to control). On the other hand, in RAW 264.7 macrophages, cell growth was stimulated by M3, M4, and M6 fractions (1.86 ± 0.136-fold increase for M3-50; 1.57 ± 0.122 and 1.65 ± 0.179 for M4-50 and M4-10, respectively, and 1.78 ± 0.021 for M6-50, compared to control). Finally, in HaCaT keratinocytes, a slight cell proliferation was observed in the presence of M3, M5, and M6 MCH fractions (1.63 ± 0.112 fold increase for M3-50; 1.38 ± 0.063 for M5-50; and 1.32 ± 0.019 and 1.35 ± 0.020 for M6-50 and M6-10, respectively, compared to control). These data clearly indicate for the first time, that not only are the marine sponge-derived MCHs biocompatible with mammalian cells and do not cause direct toxicity, but also that the positive effects on proliferation could be exploited for cosmetic or regenerative medicine purposes.

### 2.3. Radical Scavenging Activity of MCH Fractions

**Figure 3:**
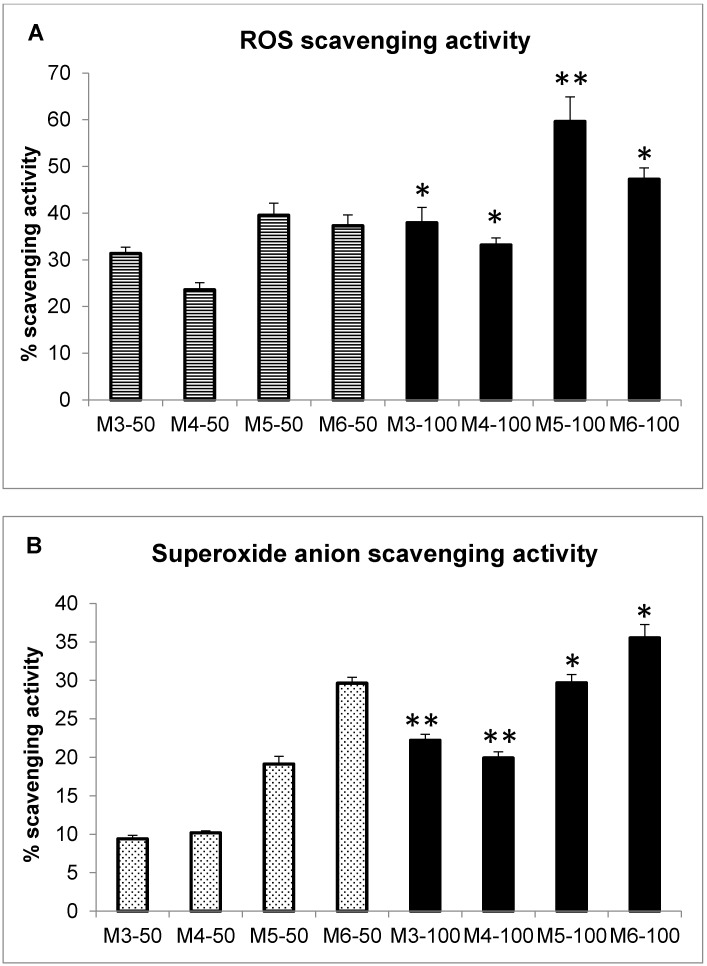
Antioxidant activity of *C. reniformis* MCHs in spectrophotometric tests. (**A**) MCH reactive oxygen species (ROS) scavenging activity by DPPH assay. Data are the mean ± S.D. of three experiments performed in duplicate, and are expressed as a percentage of antioxidant activity with respect to the absorbance of the negative control (calculated as specified in Methods (Section 4.6)). Striped bars: MCH concentration 50 g/mL; black bars: MCH concentration 100 g/mL. Asterisks indicate significance in a paired Tukey test between the same MCH fraction at 100 g/mL and at 50 g/mL concentration (* *p* < 0.05, ** *p* < 0.005, respectively). (**B**) MCH superoxide scavenging activity by Nitro Blue Tetrazolium (NBT)/riboflavin assay. Data are the mean ± S.D. of three experiments performed in duplicate, and are expressed as a percentage of antioxidant activity with respect to the absorbance of the negative control (calculated as specified in the Methods (Section 4.7)). Dotted bars: MCH concentration 50 g/mL; black bars: MCH concentration 100 g/mL. Asterisks indicate significance in a paired Tukey test between the same MCH fraction at 100 g/mL and at 50 g/mL concentration (* *p* < 0.05, ** *p* < 0.005, respectively).

Since the antioxidant properties of marine collagen peptides both from invertebrate and vertebrate sources have been well-documented in the last decade [36,42,50,51,52,53,54,55], we also decided to assess the radical scavenging activity of the four MCH fractions, both in cell-free and cellular in vitro tests to evaluate their potential as antioxidant drugs/supplements. In particular, for the cell-free measurements, two spectrophotometric tests were employed: the DPPH radical scavenging activity test for generic reactive oxygen species (ROS), and the Nitro Blue Tetrazolium (NBT)/riboflavin test measuring superoxide anion scavenging activity (Figure 3). All four MCH fractions showed generic ROS scavenging activity by the DPPH test that was 23% higher than the negative control at concentrations of 50 µg/mL and 100 µg/mL (Panel A, striped bars and black bars, respectively). In particular, the fractions with the lowest activity were M3 and M4, whose values ranged from 31.3% to 37.9% for M3, and from 23.6% to 33.2% for M4, at 50 and 100 µg/mL, respectively. Conversely, the most effective fractions were M5 and M6, which showed an increase in scavenging activity of 39.5% and 37.3% at 50 µg/mL concentration (striped bars) for M5, and 59.6% and 47.2% at 100 µg/mL for M6, respectively (black bars). Similar chemical behavior was also observed in the NBT/riboflavin test, where the ability to scavenge the superoxide anion was measured again for the MCP fractions at concentrations of 50 µg/mL and 100 µg/mL (Panel B, pointed bars and black bars, respectively). Also, in this case, M3 and M4 fractions showed the lowest scavenging activity, especially at 50 µg/mL concentration, which was slightly below 10% for both (dotted bars); while at 100 µg/mL, the increases were 22.2% for M3, and 19.9% for M4 (black bars). Again, M5 and M6 showed the highest scavenging values, ranging from 19.1% to 29.7% for M5, and from 29.2% to 35.5% for M6, at 50 and 100 µg/mL concentrations, respectively. Our results regarding the ROS scavenging activity analyzed by spectrophotometric tests often showed a higher performance with respect to a number of papers analyzing the scavenging activity by the very same analytical tests in marine collagen peptides from fishes [51,52,53,54,55], molluscs [50], and jellyfishes [36,42]. Indeed, in these papers, percentages of scavenging activity in the same range of our results are observable (≈20–70% scavenging activity). In some cases, peptide concentrations were similar to ours [50,52], with a DPPH scavenging activity in the range of 10–25% for squid-derived peptides [50] and 10–40% for crocein croaker-derived peptides [52]. However, significantly much higher peptide concentrations were used in other cases (from 500 µg/mL to 1 mg/mL), and obtained lower performances with respect to ours by DPPH scavenging assay [53,55]. One example is the case of peptides from shark collagen, which demonstrated a scavenging activity of 19–22% at a concentration of 500 µg/mL [53], or for peptides from Nile Tilapia [55], resulting in a scavenging activity of 10–25% at a concentration of one mg/mL. Thus, these data indicate a much better performance of the MCH fractions from *C. reniformis* with respect to other marine sources in the antioxidant activity. Since it has been demonstrated that *C. reniformis* collagen is significantly more glycosylated by post-translational modifications [49] with respect to the other collagens in the Metazoa, the presence of sugar moieties on the peptides deriving from the enzymatic hydrolysis could enhance the antioxidant activity with respect to collagen hydrolysates from other marine sources.

**Figure 4:**
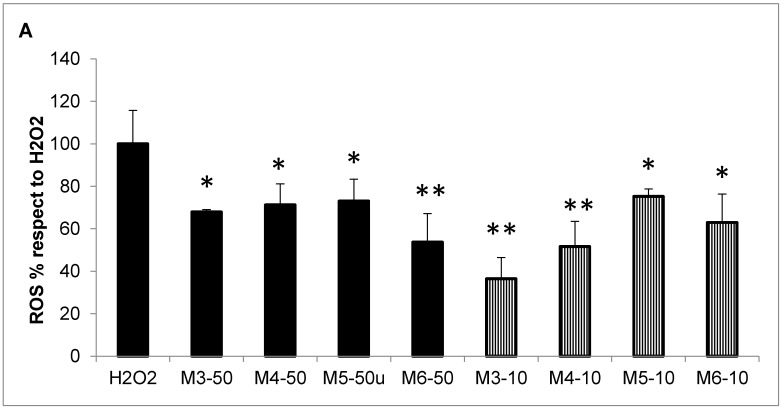

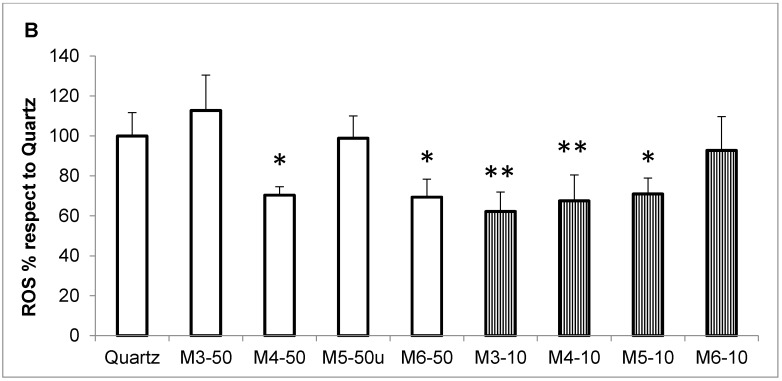
*C. reniformis* MCH ROS scavenging activity in in vitro assays. (**A**) Intracellular ROS production measured by H2DCF-dA (2′,7′-dichlorodihydrofluorescein diacetate) fluorimetric analysis in RAW 264.7 murine macrophages incubated for two hours with 200 µM of H_2_O_2_ (positive control) in the presence or absence of 50 µg/mL (black bars) or 10 µg/mL (striped bars) of MCH fractions. Results are expressed as percentages of ROS production with respect to the positive control (H_2_O_2_), and are the mean ± SD of three experiments performed in quadruplicate. Asterisks indicate significance in Tukey test (ANOVA *p* < 0.0001; Tukey vs. H_2_O_2_, * *p* < 0.05, ** *p* < 0.005, respectively). (**B**) Intracellular ROS production in RAW 264.7 cells incubated for two hours with 100 µg/mL of quartz (positive control) in the presence or absence of 50 µg/mL (white bars) or 10 µg/mL (striped bars) MCH fractions. Results are expressed as percentages of ROS production with respect to the positive control (quartz), and are the mean ± SD of three experiments performed in quadruplicate. Asterisks indicate significance in Tukey test (ANOVA *p* < 0.005; Tukey vs. quartz, * *p* < 0.05, ** *p* < 0.01, respectively).

The choice of the DPPH assay was done because is the most used method to measure the ROS scavenging activity of collagen hydrolysates [50,51,52,53,54,55], which allowed us to compare our results to those of other papers in the field. Notwithstanding, there are some drawbacks in this method, i.e., it does not enable discriminating between hydrogen atom transfer and single electron transfer mechanisms, and could fail in measuring peroxyl radical scavenging ability [56]. Thus, to overcome these limits, and in order to assess the antioxidant activity in a more physiological setting, we measured the intracellular scavenging activity of the four MCP fractions also in an activated macrophage cellular model by use of the 2′,7′-dichlorodihydrofluorescein diacetate (H_2_DCF-dA) fluorescent probe. Indeed, this is the same probe used in the fluorimetric cell-free ORAC (Oxygen Radical Absorbance Capacity) test, which is considered an accurate and physiologically relevant method for measuring the antioxidant capacity. In detail, the inhibition of ROS production was measured in stimulated RAW 264.7 murine macrophages by the use of an H_2_DCF-dA in vitro assay. Cells were challenged with 200 µM of hydrogen peroxide or with 100 µg/mL crystalline silica (quartz, mean microparticle size below five µm) in the presence or absence of the four MCH fractions (50 µg/mL and 10 µg/mL). After two hours of incubation, the intracellular ROS production was quantified by the use of the ROS-sensitive fluorescent probe (Figure 4). Both stimuli significantly enhanced ROS production in RAW macrophages by 334 ± 28.1% in the case of hydrogen peroxide and by 201 ± 39.5% in the case of quartz with respect to untreated cells. The inhibition of ROS production by the different MCH fractions was then expressed as the percentage with respect to the two positive controls, hydrogen peroxide (H_2_O_2_, panel A) and quartz (Quartz, panel B). All of the MCH fractions were able to inhibit, even if by different degrees of efficiency, both hydrogen peroxide and quartz-induced ROS in RAW macrophages. In particular, in the hydrogen peroxide-stimulated samples (Figure 4A), all of the MCH fractions significantly inhibited ROS production both at the concentration of 10 µg/mL (striped bars, 63.5% inhibition for M3, 48.4% for M4, 24.7% for M5, and 37% for M6, respectively, compared to H_2_O_2_), as well as at 50 µg/mL (black bars, 32%, 28.7%, 26.9%, and 46.2% inhibition for M3, M4, M5, and M6, respectively, compared to H_2_O_2_). For what concerns the quartz stimulation, the significant inhibition of ROS production was obtained by the use of M4 and M6 MCH fractions at the concentration of 50 µg/mL (Panel B, white bars, 29.6% and 30.6% inhibition, respectively, compared to quartz) and by M3, M4, and M5 MCH fractions at 10 µg/mL (striped bars, 37.8%, 32.5% and 29% inhibition, respectively, compared to quartz). Since the ROS that were measured in these experiments are indeed intracellular species produced in activated cells, the antioxidant activity demonstrated by the *C. reniformis* MCH fractions is even more significant. In fact, it means that these peptides are able to be at least partially internalized by cells, and are able to act directly in the cell cytoplasm, with the result of quenching the potentially dangerous respiratory burst in the activated cells of the immune system. Overall, these results open the possibility of using *C. reniformis* MCH fractions as antioxidant drugs through systemic, oral, or cosmetic supplementations.

### 2.4. Effect of MCH Fractions on Fibroblast Collagen Expression and Release

**Figure 5:**
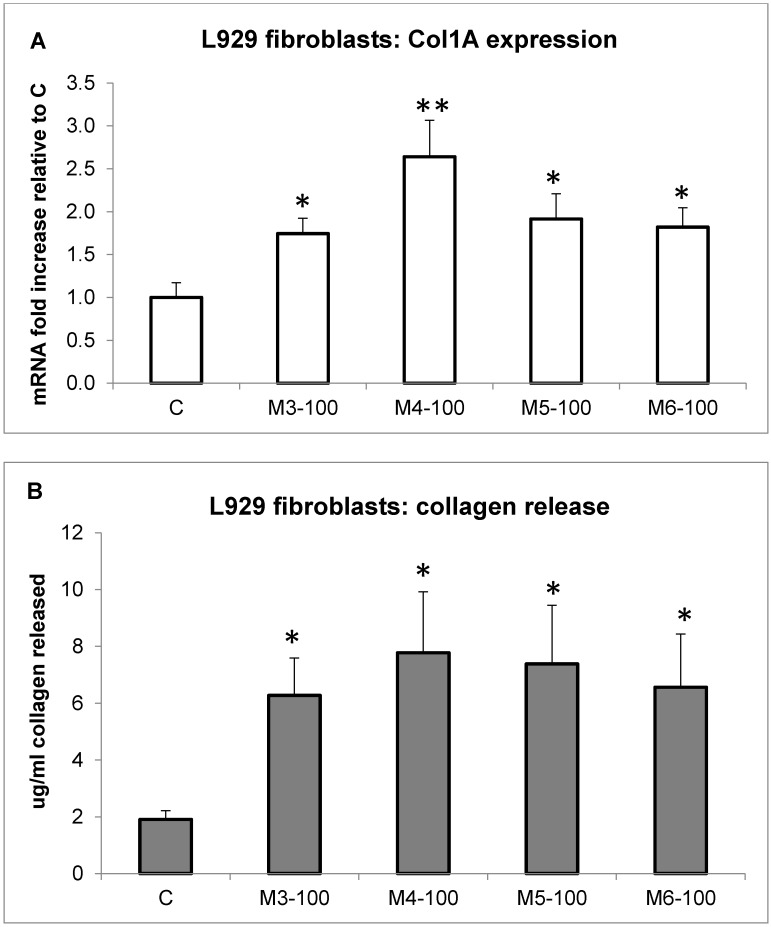
MCH-stimulated collagen gene expression and release. (**A**) L929 fibroblast gene expression measured by qPCR analysis of collagen 1A after 24 h of incubation with 100 µg/mL of the four MCH fractions. Data are normalized on the ubiquitin housekeeping gene, and expressed as an mRNA fold increase compared to control cells. Results are the mean ± SD of three experiments performed in triplicate. Asterisks indicate significance in Tukey test (ANOVA *p* < 0.0001, Tukey vs. C, * *p* < 0.05, ** *p* < 0.005, respectively). (**B**) Colorimetric collagen quantification by Sircol assay in the cell medium of L929 fibroblasts incubated in the same conditions as (A). Results are the mean ± SD of three experiments performed in duplicate. Asterisks indicate significance in Tukey test (ANOVA *p* < 0.001, Tukey vs. C, * *p* < 0.05).

Quite recently, it has been reported that collagen and/or collagen hydrolysates from various marine sources are able to stimulate collagen deposition in higher organisms [11,42,45,46]. Thus, we tested in vitro, by molecular studies, the hypothesis regarding whether the MCH fractions obtained from *C. reniformis* were able to stimulate collagen deposition in fibroblasts. In synthesis, the four peptide fractions were added to L929 murine fibroblasts for 24 h to evaluate their effect on collagen 1A (Col1A) expression and release. Indeed, all MCH fractions (100 µg/mL concentration) significantly enhanced Col1A mRNA expression and protein release in the cell medium (Figure 5). By qPCR analysis, it was possible to observe an mRNA expression fold increase of 1.74 ± 0.18, 2.64 ± 0.42, 1.91 ± 0.294, and 1.82 ± 0.22 for the M3, M4, M5, and M6 fractions, respectively, compared to the control (Panel A). Similarly, the protein release in the cell medium was quantified by Sircol assay, and the results of collagen production (Panel B) parallel the qPCR analysis. In detail, the fold increase of collagen release in the fibroblast cell medium was of 3.3 ± 0.68, 4.1 ± 1.12, 3.8 ± 1.08, and 3.4 ± 0.97 for the M3, M4, M5, and M6 fractions, respectively, compared to control. From these results, we can infer that the M4 fraction seems to be the most efficient.

We can conclude that in the fibroblast cellular model, the *C. reniformis* MCHs are not only able to promote cell proliferation (Figure 2), but also stimulate collagen deposition with a regulation of the gene at the transcriptional level. These characteristics, together with the significant antioxidant activity (Figure 4) of these peptides, could be very well employed in cosmetic formulations for anti-aging treatments. Since it is quite uncommon to have single molecular species with such heterogeneous and exploitable biological activities, as in the case of *C. reniformis* MCHs, and on the contrary, cosmetic formulations usually are a concoction of many different compounds, the possibility of using a single component that exerted all the necessary actions on aged and wrinkled skin would be an added value in those formulations. Indeed, in vivo results from a cosmetic formulation containing *C. reniformis* (non-digested) collagen extracts have already demonstrated their usability on human skin [24], obtaining results comparable to conventional mammalian collagen formulations by the evaluation of classical skin parameters (pH, hydration, sebum production). Starting from our promising in vitro results, hopefully, we will perform similar experiments in the future in an in vivo setting with the possibility of comparing the two *C. reniformis* collagen formulations (i.e., non-digested and digested) and definitely assessing the advantages and disadvantages of each.

### 2.5. Effect of MCP Fractions on UV-Induced Cell Death and Gene Expression

**Figure 6:**
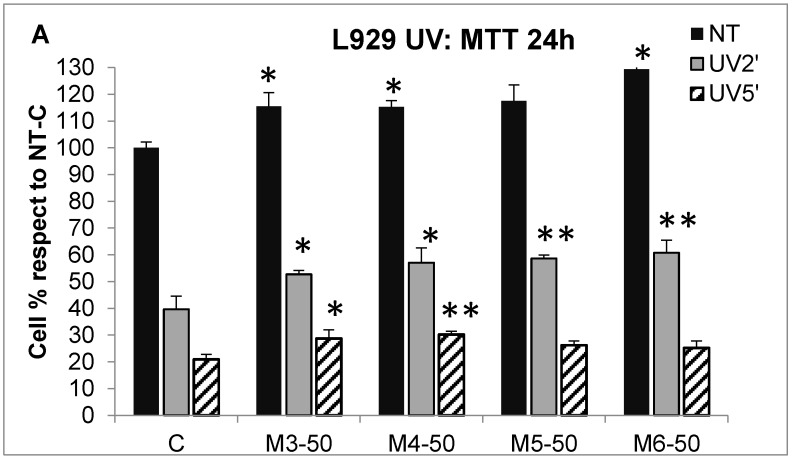

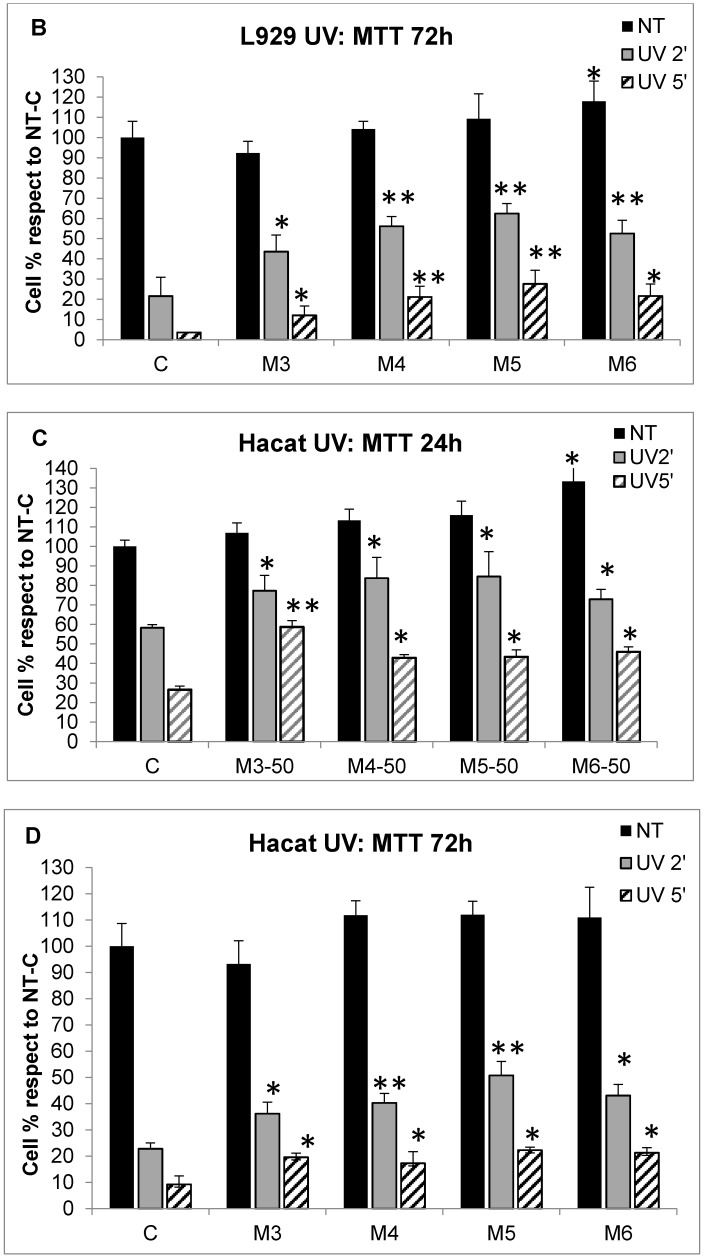
MCH cell death rescue after UV radiation. (**A**) Cell death evaluation by the MTT test at 24 h in L929 fibroblasts after UV radiation for two minutes and five minutes (corresponding to total radiation doses of 90 mJ/cm^2^ and 227 mJ cm^2^, respectively) in the presence or absence of 50 µg/mL MCH fractions. Black bars, untreated cells; grey bars, cells irradiated for two minutes; striped bars, cells irradiated for five minutes. Results are expressed as cell percentage compared to control cells (NT-C bar), and are the mean ± SD of three experiments performed in quadruplicate. Asterisks indicate significance in Tukey test (ANOVA *p* < 0.001, Tukey vs. the respective C, * *p* < 0.05, ** *p* < 0.01). (**B**) L929 in the same conditions as (A) evaluated at 72 h. Asterisks indicate significance in Tukey test (ANOVA *p* < 0.0001, Tukey vs. the respective C, * *p* < 0.05, ** *p* < 0.01). (**C**) Cell death evaluation by the MTT test at 24 h in HaCaT keratinocytes after UV radiation for two minutes and five minutes in the presence or absence of 50 µg/mL MCH fractions. Black bars, untreated cells; grey bars, cells irradiated for two minutes; striped bars, cells irradiated for five minutes. Results are expressed as cell percentage compared to control cells (NT-C bar), and are the mean ± SD of three experiments performed in quadruplicate. Asterisks indicate significance in Tukey test (ANOVA *p* < 0.01, Tukey vs. the respective C, * *p* < 0.05, ** *p* < 0.01). (**D**) HaCaT cells in the same conditions as (C) evaluated at 72 h. Asterisks indicate significance in Tukey test (ANOVA *p* < 0.01, Tukey vs. the respective C, * *p* < 0.05, ** *p* < 0.01).

Another recently investigated property of collagen and collagen hydrolysates from marine sources is their photoprotective effect on UV-damaged skin cells, which has already shown some promising in vivo results [42,57,58]. Thus, also for our sponge collagen hydrolysate fractions, the effect in favoring cell survival was evaluated in UV-challenged fibroblasts and keratinocytes in order to assess their potential use as photoprotective agents in cosmetic assets (Figure 6). L929 fibroblasts (panels A–B) and HaCaT keratinocytes (panels C–D) were flashed with a UV bulb light for two minutes and five minutes (corresponding to total radiation doses of 90 mJ/cm^2^ and 227 mJ cm^2^) in the presence or absence of 50 µg/mL MCP fractions, and cell viability was then measured after 24 h or 72 h by the MTT test (panels A/C, and panels B/D, respectively). Results are expressed as percentages of cell survival with respect to untreated, control cells (black bars, C sample) both at 24-h and 72-h end points. At 24 h (panel A), L929 fibroblasts showed a slight but significant increase in cell number in untreated, not-UV flashed cells, after incubation with M3, M4, and M6 fractions (black bars, 15.5%, 15.3%, and 29.3% cell number increase compared to C, respectively). In the same panel, after the two UV doses, an increased cell survival was also observed in the cells incubated with the four MCHs, even if at different rates. In particular, at the lowest UV dose (grey bars, UV 2′) a higher and significant cell survival was guaranteed by all four MCH fractions with respect to the control cells (13.1%, 17.4%, 19.1%, and 21.2% cell number increase for the M3–M6 fraction, respectively), while at the highest UV dose (striped bars, UV 5′), only the M3 and M4 fractions were still able to slightly increase cell survival compared to the control (8.1% and 10.2% cell increase, respectively). On the other hand, at 72 h (panel B), since the control, which were untreated cells (black bars) in these conditions, had reached confluence, it was not possible to observe any cell number increase after incubation with the four MCH fractions alone without UV treatment. Conversely, all four fractions were able to ensure a greater cell number survival after the two UV-dose treatments. In particular, at the lowest UV dose (grey bars, UV 2′), the increased rates of survival, compared to the control, were 22.0%, 34.6%, 40.8%, and 30.9% for M3–M6 fraction, respectively; while at the highest UV dose (striped bars, UV 5′), the increased cell survival rates were 8.4%, 17.5%, 24.0%, and 17.9% for M3–M6 fractions compared to the control, respectively.

The positive effect on cell survival after UV treatment was also observed in HaCaT keratinocytes in the presence of the four MCH fractions both at 24-h and 72-h end points (panels C and D, respectively). In particular, at 24 h (panel C), a significant increase in cell number was observed even in untreated, not-UV flashed cells (black bars) incubated with the M6 fraction, compared to the control (33.3% cell number increase). On the other hand, in the same panel, all four MCH fractions were able to enhance cell survival after the two UV-dose treatments. In particular, at the lowest UV dose (grey bars, UV 2′), the increased rates of survival were of 18.9%, 25.4%, 26.2%, and 14.6% for M3–M6 compared to the control, respectively; while at the highest UV dose (striped bars, UV 5′), the increased cell number percentage was of 32.1%, 16.3%, 16.8%, and 19.4% for M3–M6 compared to the control, respectively. Similarly, at the 72-h end point (panel D), the increased survival rates at the lowest UV-dose (grey bars, UV 2′) were 13.4% 17.4%, 27.9%, and 20.3% for M3–M6 compared to the control, while for the highest UV dose (striped bars, UV 5′), the enhanced cell number percentage was 10.3%, 8.1%, 13.0%, and 12.1% for M3–M6 treated cells compared to the control, respectively. As observed in L929 cells, as well as for HaCaT keratinocytes at the 72-h end point, it was not possible to observe any increase in the number of cells incubated with the four MCHs alone without UV treatment (black bars), which was again due to all the samples reaching confluence in these conditions.

Overall, these results demonstrate a clear beneficial effect regarding the cell survival of all four *C. reniformis* MCH fractions in UV-damaged skin cell cultures, with the M4 and M5 fractions showing the most performing features. From the data collected until now, we can infer that the rescuing abilities are probably due to a combination of effects between the antioxidant properties of the sponge collagen peptides together with the cell growth-promoting abilities that have been demonstrated in this paper (Figure 2, Figure 3 and Figure 4).

**Figure 7:**
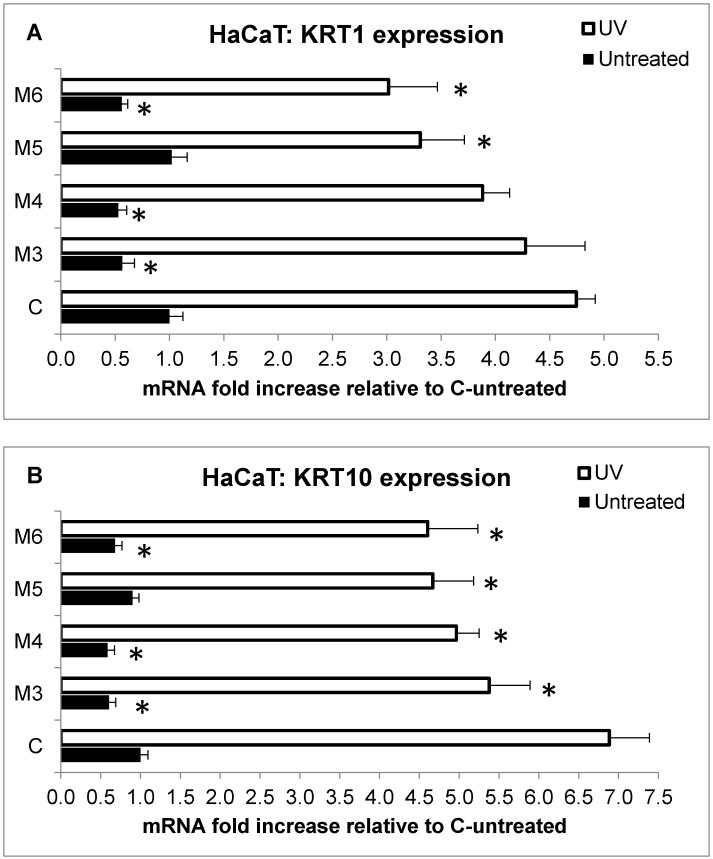
Gene expression of keratins in UV-radiated HaCaT keratinocytes. (**A**) Keratin 1 (KRT1) gene expression after 24 h measured by qPCR analysis in HaCaT keratinocytes irradiated (white bars) or not (black bars) by UV for two minutes in the presence or absence of 50 g/mL MCH fractions. Data are normalized on the ubiquitin housekeeping gene and expressed as an mRNA fold increase compared to the control, which was untreated cells (C, black bar), and are the mean ± SD of three experiments performed in triplicate. Asterisks indicate significance in Tukey test (ANOVA *p* < 0.00001, Tukey vs. the respective C, * *p* < 0.05). (**B**) Keratin 10 (KRT10) gene expression in HaCaT keratinocytes in the same conditions as (A). Asterisks indicate significance in Tukey test (ANOVA *p* < 0.00001, Tukey vs. the respective C, * *p* < 0.05).

Finally, the gene expression profile of two important genes that are highly overexpressed in UV-stressed keratinocytes and are responsible for skin thickening and loss of elasticity [59], namely keratin 1 and 10 (KRT1 and KRT10), were analyzed by qPCR in HaCaT cells. The results are displayed in Figure 7, and show the expression profile at 24 h of the two keratin genes (KRT1 in panel A and KRT10 in panel B, respectively) in cells incubated with the four MCHs alone (black bars) or after a two-minute UV treatment (corresponding to a 90 mJ/cm^2^ total radiation dose, white bars). M3, M4, and M6 fractions were able to inhibit both KRT1 and 10 mRNA expression already in untreated, not-UV challenged cells compared to the control (black bars, both panels). In particular, the KRT1 gene (panel A) underwent mRNA decreases of 43%, 47%, and 44% in the presence of M3, M4, and M6 fractions, respectively, compared to the control (C-untreated); meanwhile, the mRNA of the KRT10 gene (panel B) diminished by 40%, 41%, and 32% in the presence of M3, M4, and M6, respectively. On the other hand, after UV challenging, all of the MCH fractions were able to inhibit KRT1 and 10 mRNA overexpression (white bars, both panels). In detail, KRT1 showed a 4.75 ± 0.174 mRNA fold increase after UV treatment compared to the untreated control (white C-UV bar, versus black C-untreated bar, panel A), while in the same conditions, KRT10 mRNA increased by 6.88 ± 0.504-fold compared to the untreated control (white C-UV bar, versus black C-untreated bar, panel B). This significant mRNA increase of the two genes after HaCaT UV-flashing was partially inhibited by the treatment with the MCH fractions. In particular, KRT1 overexpression was inhibited by M5 and M6 fractions alone (Panel A, white bars, 1.44 and 1.73-fold decrease compared to C-untreated, respectively), while KRT10 overexpression was inhibited by all of the MCH fractions, even if with different rates (Panel B, white bars, 1.52, 1.92, 2.21, and 2.28-fold decreases for M3–M6 compared to C-untreated, respectively). Overall, these data indicate a real therapeutic effect of the four MCH fractions on UV-stressed skin cells. Indeed, *C. reniformis* MCHs, through their properties, can act on the skin in different ways by (i) promoting a partial rescue from UV-induced cell death (Figure 7), which is likely thanks to the ROS scavenging activity (Figure 3 and Figure 4) and the cell growth stimulation (Figure 2), (ii) reducing the inflammatory response of immune cells recruited in the damaged skin, again by its ROS scavenging activity, and (iii) by counterbalancing the stress molecular responses of keratinocytes to UV radiation such as the increase of keratin production and deposition that contribute to photoaging through loss of elasticity and skin thickening. Finally, if we add to these already important effects also the stimulation of collagen production in fibroblasts (Figure 5), we can conclude that indeed, cosmetic and pharmacological formulations for aged and photodamaged skin repair could really benefit from the presence of *C. reniformis*-derived MCHs to enhance their efficacy.

### 2.6. Effect of MCP Fractions on Wound Healing

**Figure 8:**
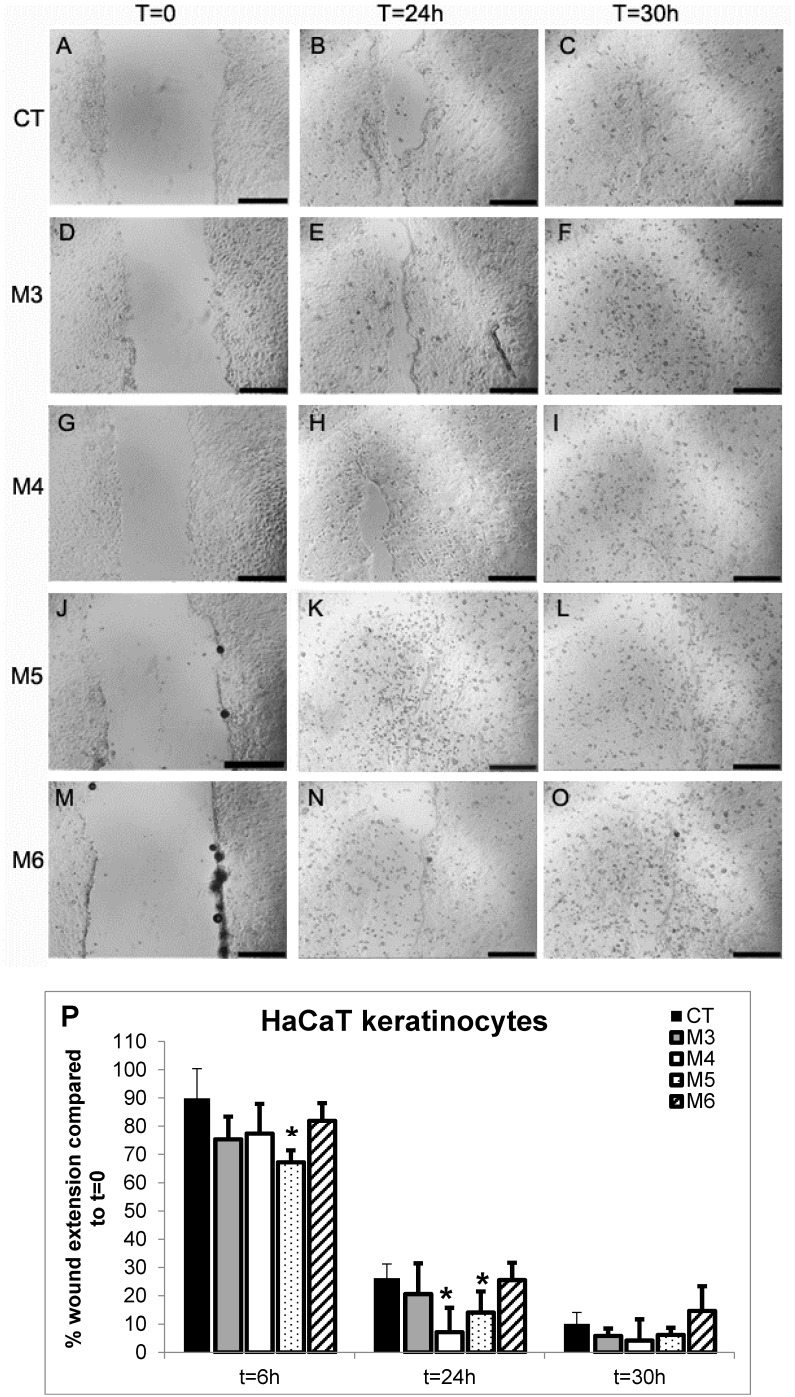
Wound-healing assay in MCH-treated HaCaT keratinocytes. (**A**–**O**) Microphotographs taken at 0 h, 24 h, and 30 h with a 4× objective of HaCaT keratinocyte monolayers during the wound-healing assay in the presence or absence of 50 µg/mL MCH fractions, in the area of the scratch made at time = 0. A–C control cells, D–F M3-treated cells, G–I M4-treated cells, J–L M5-treated cells, and M–O M6-treated cells. Black bars span 50 µm. (**P**) Quantitative evaluation of the wound-healing degree of HaCaT cells over time. To determine the degree of wound healing, the closing distance of the scratch was measured two times in each photograph by using the ImageJ program free software (http://imagej.nih.gov/ij/). Data are expressed as percentages of the closing distance of each sample with respect to the same sample at time = 0. Experiments were repeated twice in quadruplicate, and data are the mean ± SD. Asterisks indicate significance in Tukey test (ANOVA *p* < 0.00001, Tukey vs. the same sample at t = 0, * *p* < 0.05).

**Figure 9:**
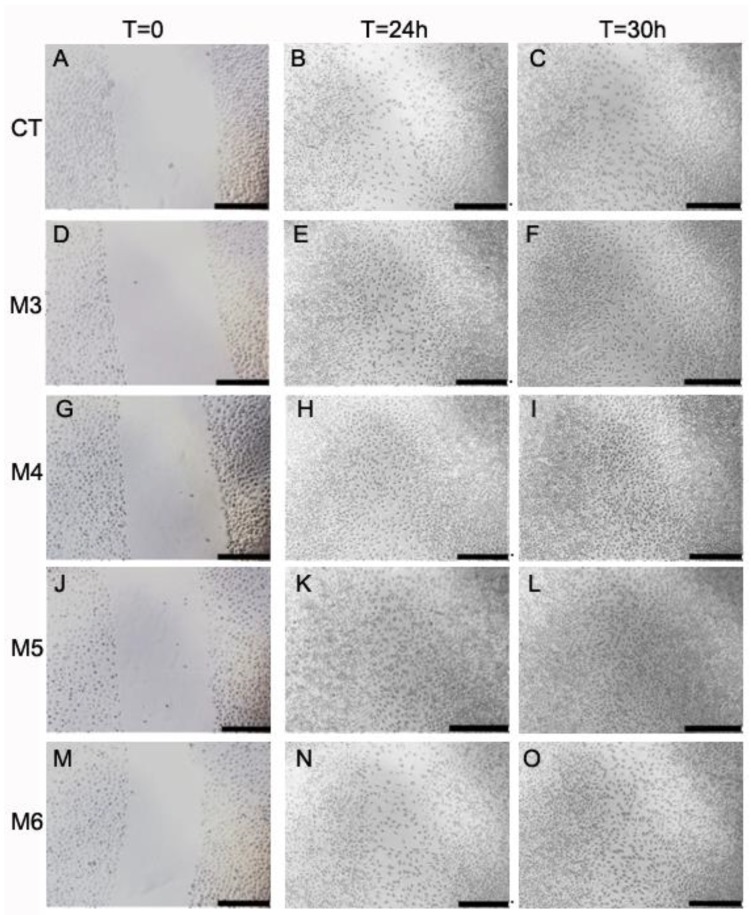
Wound-healing assay in MCH-treated L929 fibroblasts. (**A**–**O**) Microphotographs taken at 0 h, 24 h, and 30 h with a 4× objective of L929 fibroblast monolayers during the wound-healing assay in the presence or absence of 50 µg/mL MCH fractions, in the area of the scratch made at time = 0. A–C control cells, D–F M3-treated cells, G–I M4-treated cells, J–L M5-treated cells, and M–O M6-treated cells. Black bars span 50 µm.

The use of marine collagen in composite biomaterials as wound dressing to enhance healing has sporadically been reported [60,61], as well as the use of marine collagen peptides alone or in combined biomaterials demonstrating wound-healing properties in vivo [40,45,62,63]. Thus, the four MCH fractions were also tested for their wound-healing properties both in HaCaT keratinocytes and in L929 fibroblasts. Cell migration/proliferation was performed by the “scratch” assay, which is an in vitro test that is widely used for these purposes [40,63,64], and is described in detail in the Materials and Methods section. The assay was performed in the presence or absence of the different MCH fractions at a concentration of 50 µg/mL. Cells were photographed at 0 h, 6 h, 24 h, and 30 h; pictures were analyzed and quantified as described in Materials and Methods, and the results are displayed in Figure 8 and Figure 9. In particular, from a qualitative point of view, in HaCaT keratinocytes (Figure 8, panels A–O) after the scratch at time = 0 (panels in the first column), it was possible to observe a progressive closure of the scratch in all of both the MCH-treated and untreated samples (panels in the second and third column corresponding to time = 24 h and = 30 h, respectively). The behavior of HaCaT cells indicates a cell proliferation by the sides of the wound gradually filling the gap that is significantly accelerated both at 6 h and 24 h by M5 treatment and at 24 h by M4 treatment with respect to the controls (CT) at the same time points, as quantified in Figure 8P (dotted bars and white bars versus black bars, respectively). In particular, for M5 treatment, the percentage of wound extension at 6 h and at 24 h was 22.7% and 15.7% less than its control, respectively (panels J–L and dotted bar in panel P), while in the case of M4 treatment, the percentage of wound extension at 24 h was 22.6% less than the control (panels G–I and white bars in panel P). Conversely, in L929 fibroblasts after the scratch at time = 0 (Figure 9, panels in the first column), it was possible to observe a progressive migration and colonization of the scratch by cells in all of the both MCH-treated and untreated samples (panels in the second and third column corresponding to time = 24 h and 30 h, respectively). The behavior of L929 fibroblasts in this case indicates first a migration of cells into the gap, and afterwards a proliferation, since it is possible to observe single cells scattered all over the scratch, even in its center, already at 24 h, while the sides of the wound are no more clearly visible, as it was indeed possible in HaCaT cells. This being the case, a qualitative wound-healing score was assigned based on the observation on increasing cell density into the scratches instead of a quantification of wound restriction, as in HaCaT analysis. Anyway, also in L929 cells, the qualitative wound-healing score assigned in blind (Table 2) revealed an increased cell migration/density in M4 and M5-treated cells both at 24 h and 30 h (second and third column, respectively) compared to the controls (CT) at the same time points. The results from both cell lines are quite comparable to those obtained by Hu et al. [40] and Ouyang et al. [63] on the same cell lines, HaCaT and L929, respectively, in both papers treated with marine collagen peptides from Nile Tilapia, with [63] and without [40] the concomitant use of chitosan. In both cases, a similar increase of wound closure was obtained at the same time and at the same concentration of peptides used by us in the two cell lines tested.

We can conclude that in both cell types, keratinocytes and fibroblasts, the M4 and M5 fractions from *C. reniformis* MCHs demonstrated promising wound-healing properties, facilitating either cell migration or proliferation at the site of the wound of epidermal and dermal cells. These fractions could be used for the treatment of such injuries, with effects comparable to those of well-known MCHs from other sources.

## 3. Conclusions

The scientific literature on the bioactive properties of MCHs from several invertebrate and vertebrate sources, as well as their use for the treatment of different types of skin injuries or in regenerative medicine assets, has been constantly growing in the last decade. This actually demonstrates the safety, as well as, in many cases, the efficacy of pharmacological, nutraceutical, and cosmetic formulations of marine collagen-derived peptides in human health issues. To date, no information about the very same bioactive properties of collagen-derived peptides in the most primitive Metazoa, namely sponges, were available. Thus, we undertook our study by choosing a marine sponge that was particularly enriched in collagen, whose chemical and bioactive properties have already been in part documented as in the case of *C. reniformis*. The collagen of this demosponge already holds promising features for its use in the production of biomaterials that are suitable for tissue engineering and regenerative medicine purposes, as has been recently demonstrated by our group. Now, with the current study, also the successful employ of peptide mixture solutions derived from its enzymatic digestion have been ascertained in different cellular models, with a particular emphasis on the antioxidant and proliferative properties that seem particularly beneficial in relieving symptoms of various skin injuries derived from UV radiation or wounding. This opens a way to the use of *C. reniformis* MCHs in drug and cosmetic formulations in in vivo studies and in humans to definitely confirm their usability. Anyway, some further studies would be necessary for that to happen, such as for example a more precise description and chemical characterization of the peptide mixtures obtained by the enzymatic digestion of the sponge collagen suspensions, possibly concentrating on the most bioactive fractions as M4 and M5 seem to be, and last but not least, a serious study on the real performances of the aquaculture systems of this marine sponge in terms of growth rates, biomass yields, and the feasibility of this type of farming to really bring such a promising product to the market.

## 4. Materials and Methods

All reagents were acquired from SIGMA-ALDRICH (Milan, Italy), unless otherwise stated.

### 4.1. Preparation of MCHs

MCHs were obtained by trypsin digestion of a collagen fibril suspension isolated from *C. reniformis,* as previously described in Pozzolini et al. 2018 [7]. Briefly, 25 g of frozen sponge tissue was minced in five volumes of 100 mM of ammonium bicarbonate, pH 8.5, and incubated overnight at 37 °C on a horizontal shaker in presence of 0.1% trypsin. Afterwards, the fluid was removed by filtration with a metallic strainer, and the solid material was suspended in three volumes of deionized water and incubated at 5 °C for three days in a rotary disk shaker. The dark and viscous suspension was then filtered with a metallic strainer, and the remained solid material was subjected to a second round of three days of water extraction. The viscous fluid was pooled and centrifuged at 1200× *g* for 10 min at 4 °C. The supernatant fluid containing the collagen suspension was then centrifuged at 12,000× *g* for 30 min at 4 °C, and finally suspended in 100 mM of ammonium bicarbonate, pH 8.5. Total protein content was evaluated by BCA (Bicinchoninic Acid assay) assay as described in Pozzolini et al. 2018 [7]. MCHs from the fibrillar collagen extract were then obtained as follows: the collagen extract was heated at 90 °C for one hour; then, it was cooled on ice, and finally trypsin digested for 18 h at 37 °C, with a 1:20 (*w*/*w*) ratio of enzyme to substrate. Subsequently, the peptide mixture was heated at 70 °C for 10 min to inactivate the enzyme, and centrifuged. The supernatant containing the MCHs was frozen and stored at −20 °C for further high performance liquid chromatography (HPLC) purification. The procedure was repeated twice.

### 4.2. SDS-Page Analysis

In order to evaluate the digestion activity of trypsin on the purified fibrillar collagen extract, MCHs were analyzed by SDS-PAGE and compared with the undigested collagen suspension; 30 μL of 1 mg/mL of each sample was mixed with 4× loading buffer (60 mM of Tris-HCl, pH 8.0, containing 25% glycerol, 2% SDS, and 0.1% bromophenol blue); then, it was boiled for 10 min and loaded on a 7.5% SDS polyacrylamide gel. The electrophoresis was carried out for two hours at 70 mA. After electrophoresis, the gel was fixed in 10% acetic acid for 30 min, and then stained for three hours with 0.05% (*w*/*v*) Coomassie blue R-250 in 15% (*v*/*v*) methanol and 5% (*v*/*v*) acetic acid. The gel was finally destained with 30% (*v*/*v*) methanol and 10% (*v*/*v*) acetic acid prior to imaging.

### 4.3. Degree of Hydrolysis (DH) Evaluation

DH was calculated as the ratio between the total amino acid content in the MCHs and the amino acid content in the undigested collagen suspension. To determine the amino acid content, the samples were previously hydrolyzed with 2 N of NaOH by autoclaving at 120 °C for 20 min. Then, they were neutralized by adding one volume of 2 N HCl and diluted 10-fold in 50 mM of NaH_2_PO_4_, pH 8. Total amino acid content was finally evaluated by adding 0.25 volumes of 8% ninhydrin solution and incubating for 10 min at 80 °C. Absorbance of each sample was read at 570 nm using a Beckman spectrophotometer (DU 640), in comparison to a L-lysine standard curve. The procedure was carried out in duplicate.

### 4.4. HPLC MCP Purification

The trypsin-digested collagen suspensions, deriving from two different extractions/digestions, were purified by preparative reversed phase high-performance liquid chromatography (RP-HPLC) to obtain the MCH fractions. A Phenomenex C18 Luna (21.2 × 250 mm) column on an Agilent series 1260 Infinity preparative HPLC separation system (Agilent Technologies Italia SpA, Milan, Italy) was used. Before the preparative purification, an analytical reversed phase HPLC was performed using a similar C18 column to establish the best gradient for the following purification. Solvent A was 0.1% formic acid in water, and solvent B was 0.1% formic acid in acetonitrile. The gradient was 0–65% B from five to 35 min, the flow rate was set to 15 mL/min, and two-minute fractions were collected monitoring the chromatogram at the two different wavelengths of 220 nm and 254 nm. All of the fractions were then concentrated under vacuum and lyophilized to remove the formic acid. The fractions were suspended at a concentration of 10 mg/mL in water to obtain the starting MCH solutions for the biological assays.

### 4.5. Hydroxyproline Content Evaluation

The hydroxyproline content was estimated by a modified method based on the chloramine-T reaction [65]. First, 0.2 mL of 1 mg/mL solution of undigested collagen fibril suspension, trypsin-digested MCP mixture, and each of the four HPLC-purified fractions were hydrolyzed with two N of NaOH by autoclaving at 120 °C for 20 min. Samples were neutralized by adding one volume of two N of HCl and then, they were diluted fourfold in deionized water. The hydroxyproline concentration evaluation was obtained by adding chloramine-T and Ehrlich’s reagent, as already described. Absorbance of each sample was read at 550 nm using a Beckman spectrophotometer (DU 640), in comparison to a cis-4–hydroxy-L-proline standard curve. The procedure was carried out in duplicate.

### 4.6. DPPH Radical Scavenging Activity

The total radical scavenging activity was evaluated by the DPPH method on each MCH fraction. The DPPH test solution, 1 mL/sample, was prepared with different amounts of the four MCH fractions (50 μg/mL and 100 μg/mL final dilution) put in 250 μL of deionized water and added to 500 μL of methanol and 250 μL of 0.2 mM of DPPH dissolved in methanol (2,2-diphenyl-1-picrylhydrazyl, Calbiochem^®^, Millipore SpA, Milan, Italy). A negative control sample containing only the DPPH test solution and a positive control sample with DPPH and 500 μg/mL ascorbic acid were prepared as well. All of the samples were incubated for 30 min at room temperature (RT) in the dark. Then, the samples were read at 517 nm using a Beckman spectrophotometer (DU 640). In the blank sample, the DPPH solution was substituted with methanol. The antioxidant activity of the MCH fractions was evaluated by the quenching of the DPPH radical using the following equation:

DPPH radical scavenging activity (%) = (A0 − A)/A0 × 100%

where A was the sample absorbance rate; and A0 was the absorbance of the negative control. The procedure was carried out in duplicate.

### 4.7. NBT Superoxide Anion Scavenging Activity

The superoxide anion scavenging activity of the MCH fractions was evaluated by the NBT/riboflavin test. Briefly, the NBT/riboflavin test solution, 1 mL/sample, was prepared with or without different amounts the four MCH fractions in duplicate (100 µg/mL and 200 µg/mL final dilution). The composition of the NBT/riboflavin test solution was the following: 15 µM of riboflavin, 500 µM of Nitro Blue Tetrazolium (NBT), 10 mM of D-L methionine, 0.025% Triton x-100, and 50 mM of phosphate buffer, pH 7.8. The blank sample was prepared as follows: 500 µM of NBT in 50 mM of phosphate buffer, pH 7.8. The samples were transferred to 3.5-cm plastic petri dishes (without cover), and then flashed for two minutes under a UV lamp (Sanikyo Denki G20T10) at a 20-cm distance (90 mJ/cm^2^ total radiation dose). Samples were then read in a Beckman spectrophotometer (DU640) at 560 nm against the blank sample. The scavenging activity (quenching of the blue color) was calculated by the same algorithm that was used for the DPPH scavenging activity.

### 4.8. Cell Cultures

The mouse macrophage cell line RAW 264.7 and the mouse fibroblast L929 cell line were obtained from the American Type Culture Collection (LGC Standards srl, Milan, Italy). The human keratinocyte HaCaT cell line (CLS Cell Lines Service, 300493) was obtained by the Cell Lines Service (GmbH, Eppelheim, Germany). Cells were cultured at 37 °C in a humidified, 5% CO_2_ atmosphere in high glucose Dulbecco’s modified Eagle’s medium (DMEM) with glutamine (Microtech srl, Naples, Italy), and supplemented with 10% fetal bovine serum (Microtech) with penicillin/streptomycin as antibiotics.

#### 4.8.1. Cell Viability

Experiments were performed in quadruplicate on 96-well plates. RAW 264.7 macrophages, L929 fibroblasts, and HaCaT keratinocytes were seeded at 5000 cells/well, and allowed to adhere overnight. Then, the four different MCH fractions (10 µg/mL and 50 µg/mL final dilutions) were added to each well, and the plates were incubated for three days at 37 °C. At the end of the experiments, cell viability was assayed by MTT test (0.5 mg/mL final concentration), as already reported [64]. Data are the means ± SD of three independent experiments performed in quadruplicate.

#### 4.8.2. ROS Detection in RAW 264.7 Cells

Experiments were performed in quadruplicate on 96-well plates as described in Scarfì et al. [66]. Briefly, RAW 264.7 macrophages were plated at a density of 25,000 cells/well, and allowed to adhere overnight. Cells were then washed once with Hank’s balanced salt solution (HBSS) and incubated for 40 min at 37 °C with 10 µM of 2′,7′-dichloro-dihydro-fluorescein diacetate (H_2_DCF-dA) dye (Life Technologies, Milan, Italy). Cell stimulation was obtained either by using 200 µM of H_2_O_2_ or 100 µg/mL of sterilized quartz particles (Q) (MIN-U-SIL 5: US Silica, Berkeley Spring Plant, SSA _BET_ = 5.2 m^2^/g) prepared as described in Scarfì et al. [67] in the presence or absence of 10 µg/mL and 50 µg/mL of the four MCHs. Enhanced ROS concentration on the Q surface was obtained by ultrasound irradiation of a 10 mg/mL sterile quartz solution (three cycles of 10 pulses each at 50 Hz) prior to addition to the cell cultures.

After incubation with the dye, cells were washed with HBSS, incubated at 37 °C for 15 min, and then challenged with 100 µg/mL of Q particles or 200 µM of H_2_O_2_ for 2 h. The plates were finally read on a Fluostar Optima BMG using 485/520 excitation/emission wavelengths. Data are the means ± SD of three independent experiments performed in quadruplicate.

#### 4.8.3. Collagen Quantification in the L929 Fibroblast Cell Medium

Collagen production by L929 fibroblasts was quantified in the cell medium by the SIRCOL^TM^ Soluble Collagen Assay (Biocolor Ltd., Carrickfergus, Northern Ireland, UK). Fibroblasts were seeded in tissue culture six-well plates at a density of 5 × 10^5^ cells/well in complete medium and allowed to adhere overnight. Then, cells were incubated for 24 h in the presence or absence of 100 µg/mL of the four MCH fractions. At the end of the incubation cell culture, media were collected, and the SIRCOL assay was performed according to the manufacturer’s instructions. Data are the means ± S.D. of three independent experiments performed in duplicate.

#### 4.8.4. UV Treatment

To evaluate cell death prevention from UV treatment by the four MCH fractions, both L929 fibroblasts and HaCaT keratinocytes were seeded in quadruplicate in 96-well plates. Both cell lines were plated at a density of 20,000 cells/well for the 24-h viability assay and at 10,000 cells/well for the 72-h assay, and allowed to adhere overnight. The four MCH fractions, at a final dilution of 50 µg/mL, were added to the wells, and then the plates were illuminated for two minutes and five minutes under an UV lamp (Sanikyo Denki G20T10) at a 20-cm distance (90 mJ/cm^2^ and 227 mJ cm^2^ total radiation dose, respectively). Cell viability was evaluated by the MTT test at 24 h and 72 h of incubation after the UV radiation, and compared to control non-irradiated samples cultured in the same conditions. Data are the mean ± SD of two independent experiments performed in quadruplicate.

#### 4.8.5. Wound-Healing Assay

To evaluate the effect of the four MCHs on cell growth/migration, the wound-healing (WH) assay was performed on HaCaT and L929 cell lines as already reported [40,63,64]. Briefly, cells were seeded on 12-well tissue culture plates at a concentration of 140,000 cells/well and incubated in complete medium for 24 h or until confluence was reached. Before plating cells, with the help of a ruler, five dots were drawn with a permanent marker on the back of the plate with correspondence to the diameter in the center of each well. The cell monolayer in each well was then scraped with a p100 pipet tip, making a straight line to create a “scratch” with the help of a ruler, and following the five black dots drawn on the back of the well. Cells were washed with PBS (Phosphate Buffered Saline) to remove detached cells and debris, and fresh medium was added in the absence or presence of the four MCH fractions at the concentration of 50 µg/mL. Then, the straight scratch line in each well was photographed in four different fixed points to the microscope at 4× objective at times: 0 h, 6 h, 24 h, and 30 h. Photos were then taken always in the same points during the time course thanks to the five dots. In fact, in each well, there were four fixed areas in between two dots. Thus, each experiment is the mean of the results of four areas in each well made in duplicate.

To determine the degree of wound healing in HaCaT cells, the closing distance of the scratch was measured two times in each photograph by using the ImageJ program free software (http://imagej.nih.gov/ij/). Data are expressed as percentages of the closing distance of each sample with respect to the same sample at time = 0. A decrease of the percentage of the closing distance indicates an increase in the growth/migration of cells. Conversely, to determine the wound-healing degree in L929 cells, a qualitative visual method was used to score the increase of cell density in the scratches of each sample over time. Photographs at the various end points were scored in blind from + to +++, indicating the increasing cell density in the area of the scratches. Data are the mean of three independent experiments performed in duplicate.

#### 4.8.6. RNA Extraction, cDNA Synthesis, and qPCR Analyses

L929 fibroblasts (5 × 10^5^), seeded the day before in complete medium in six-well plates, were incubated with 100 µg/mL of the four different MCH fractions for 24 h, and then, expression of collagen 1A (Col1A) was quantified by qPCR respect to control, untreated cells. HaCaT keratinocytes (5 × 10^5^) that were seeded the day before in complete medium in six-well plates, were challenged with 50 µg/mL of the four different MCH fractions, UV-irradiated for two minutes as already described, and then, the expression of Keratin 1 (KRT-1) and Keratin 10 (KRT-10) was quantified by qPCR after 24 h with respect to control untreated cells. Total RNA was extracted using the RNeasy Mini Kit (Qiagen, Milan, Italy) according to the manufacturer’s instructions. The quality and quantity of RNA was analyzed using a NanoDrop spectrophotometer (Nanodrop Technologies, Wilmington, DE, USA). The cDNA (1 µg per sample for the L929 cells and 500 ng for the HaCaT cells) was synthesized by using an iScript cDNA Synthesis Kit (Bio-Rad Laboratories, Milan, Italy). Each PCR reaction was performed in 10 μL containing: 1× master mix iQ SYBR^®^Green (Bio-Rad), 0.2 µM of each primers, and 5 ng of synthesized cDNA. All of the samples were analyzed in triplicate. The following thermal conditions were used: initial denaturation at 95 °C for three minutes, followed by 45 cycles with denaturation at 95 °C for 15 s, and annealing and elongation at 60 °C for 60 s. The fluorescence was measured at the end of each elongation step. Values were normalized to ubiquitin (reference gene) mRNA expression both for L929 mouse fibroblasts and HaCaT human keratinocytes. All of the primers (Table 3) were designed using the Beacon Designer 7.0 software (Premier Biosoft International, Palo Alto, CA, USA) and obtained from TibMolBiol (Genova, Italy). Data analyses were obtained using the DNA Engine Opticon^®^ 3 Real-Time Detection System Software program (3.03 version), and in order to calculate the relative gene expression compared to an untreated (control) calibrator sample, the comparative threshold Ct method [68] was used within the Gene Expression Analysis for iCycler iQ Real Time Detection System software (Bio-Rad) [69]. Data are means ± SD of two independent experiments performed in triplicate.

### 4.9. Statistical Analysis

Statistical analysis was performed using one-way ANOVA plus Tukey’s post-test (GraphPad Software, Inc., San Diego, CA, USA). *P* values < 0.05 were considered significant.

## Figures and Tables

**Table 1:** Hydroxyproline content in the various samples before and after HPLC fractionation. Hydroxyproline content in µg/mL measured by the chloramine-T assay (see Methods (Section 4.6)) in the *C. reniformis* undigested collagen suspension (CS), in the trypsin-digested collagen solution (Total MCP), and in the four HPLC-purified hydrolysate fractions (M3–M6). Data are expressed as the mean ± S.D. of three independent experiments performed in duplicate.

	µg/mL Hyp
undigested CS	83.21 ± 12.3
Total MCPs	109.99 ± 4.78
M3	7.04 ± 0.32
M4	30.40 ± 6.72
M5	37.23 ± 3.56
M6	16.76 ± 4.51

**Table 2:** Wound-healing score in L929 cells, in the presence or absence of MCHs, obtained by a qualitative visual method to quantify the increase of cell density in the scratches of each sample during time. Photographs at the various end points were scored in blind from + to +++, indicating the increasing cell density in the area of the scratches. Data are the mean of two experiments performed in quadruplicate.

P	Wound Healing Score in L929 Fibroblasts	
	24 h	30 h
CT	+	++
M3	++	++
M4	++	+++
M5	+++	+++
M6	+	++

**Table 3:** Primer sequences used in the qPCR analyses. Primer pairs used for the qPCR experiments in gene expression quantification of murine collagen 1A (Col 1A MM), murine, and human ubiquitin (ubiquitin MM and HS, respectively), human Keratin 1 and 10 (keratin 1 HS and 10 HS, respectively).

GENE	GenBank	Forward	Reverse
**Col1A MM**	NM_007742.4	5′-CTgCTggTCCTgCTggTC-3′	5′-CCTTgTTCgCCTgTCTCAC-3′
**Ubquitin MM**	NM_019639	5′-GACAGGCAAGACCATCAC-3′	5′-TCTGAGGCGAAGGACTAAG-3′
**Keratin 1 HS**	NM_006121.3	5′AAgCCACACCACCATCAg-3′	5′CACCTCCAgAgCCATAgC-3′
**Keratin 10 HS**	NM_000421.3	5′-CCgAgTgCCAgAATACTgAATACC-3′	5′-TAgCCgCCgCCgAAACTTC-3′
**Ubiquitin HS**	NM_021009.6	5′-ATTTgggTCgCAgTTCTTg-3′	5′TgCCTTgACATTCTCgATggT-3′
